# The impact of patient engagement on patient safety in care transitions after cancer treatment: Protocol for a systematic review and meta-analysis

**DOI:** 10.1371/journal.pone.0307831

**Published:** 2024-08-27

**Authors:** Larissa Brust, Ingo Schmidt-Wolf, Matthias Weigl

**Affiliations:** 1 Institute for Patient Safety (IfPS), Medical Faculty, University Hospital Bonn, Bonn, Germany; 2 Center for Integrated Oncology (CIO), Medical Faculty, University Hospital Bonn, Bonn, Germany; Haile Selassie I Hospital: Yekatit 12 Hospital Medical College, ETHIOPIA

## Abstract

**Background:**

Transitions of care after cancer treatment pose a major challenge for patient safety as adverse events and unplanned healthcare utilization occur frequently. At this point, patient and family engagement (PFE) is particularly valuable since patients and their families experience various challenges along this pathway, such as changing roles and recurrent needs to navigate across structural gaps between different services. However, there is currently a lack of evidence on the impact of PFE on patient safety in transitions after cancer treatment.

**Objective:**

To systematically review and synthesize evidence on effects of different PFE interventions on patient safety in the transition of care after cancer treatment.

**Methods:**

This protocol for a systematic review with meta-analysis follows PRISMA-P guidelines. A comprehensive database search will be conducted in MEDLINE, EMBASE, CENTRAL, CINAHL, and APA PsycInfo. Trial registries and grey literature will be searched, forward and backward citation tracking will be performed. Trials with prospective, longitudinal, interventional study designs will be included if they evaluate PFE interventions on patient safety outcomes (primary outcomes: healthcare utilization, patient harm, adherence, patient experience; secondary: quality of life, distress); eligible studies need to survey patients with any oncological disease during or after transition following cancer treatment. Results will be synthesized narratively and meta-analytically using a random-effects model. Risk of bias will be assessed using the Cochrane RoB-2 and revised JBI critical appraisal tool. The certainty of evidence will be judged according to the GRADE approach.

**Discussion:**

Robust evidence of effectiveness is needed to establish PFE interventions for patient safety in care transitions for oncological patients. This review will allow evidence-based conclusions about types and effects of different PFE interventions for transitional safety in oncology care and inform stakeholders in designing sustainable PFE activities.

**Trial registration:**

PROSPERO (CRD42024546938), OSF (doi.org/10.17605/OSF.IO/9XAMU).

## Introduction

Transitions of care (ToC) are a significant challenge for patient safety as unexpected and preventable adverse events and unplanned healthcare utilization occur frequently [1–3]. In particular, patient transitions across the cancer care continuum (i.e., detection, diagnosis, treatment, survivorship) pose a major challenge [4, 5]. In terms of transitions within or after cancer treatment, high rates of potential harm have been reported: such as 11–21% (disease-specific) readmissions [6–8], significant difficulties with adherence to treatment regimens [9], physical and emotional challenges, patient dissatisfaction, and high costs [5, 10–13].

In this paper, we focus therefore on ToC after completion of an active cancer treatment (e.g. chemotherapy, radiotherapy, surgery). This treatment may be followed by subsequent complimentary interventions (e.g. radiotherapy after surgery), follow-up care (including psychosocial support, reintegration into family and professional life, and detection of health problems [14]), or no care. Transitioning between different specialties and crossing structural gaps between different services is particularly challenging for patients and their families since they are recovering from an acute event with often intense treatment, and they are then additionally confronted with shifting roles and responsibilities, e.g., when healthcare professionals change or the agency and responsibility for one’s own health return predominantly to the patient [15–18]. Patient and family engagement (PFE) has been thus proposed as a key strategy to improve patient safety in ToC [16, 19–21].

Specifically, PFE has been identified as a key approach to improving patient safety [22–27] and is defined as the collaboration of patients, families, their representatives, and health professionals in an active partnership to improve health and health care [28–30]. PFE can be conveyed along a continuum of types, depending on how active the patient’s role is conceived (i.e., consultation, involvement, partnership and shared leadership) [30, 31]. Moreover, various PFE forms can be distinguished according to different levels across the health care system (i.e., direct care, organizational, policy making) [30]. The underlying program theories are complex [32, 33], but patients have a broad perspective on healthcare through their diverse experiences and can therefore make an important contribution to patient safety [32, 34]. Therefore, the World Health Organization strongly recommends PFE in its Global Patient Safety Action Plan as a key strategic aim [35].

While the value of PFE for improving patient safety is widely acknowledged, there is actually limited knowledge about its role and effects in preventing or reducing harm and healthcare utilization during ToC [36–40]. Furthermore, little attention has been paid to the different types and levels of PFE—especially on the organizational level [22, 41–43]. These shortcomings are especially pronounced for the ToC of oncological patients [43, 44]. To the best of our knowledge, there is currently no systematic review with meta-analysis on the overall effects of PFE on patient safety in care transitions after cancer treatment.

It is important to address these gaps in our knowledge base since care designed around specialist divisions of patient care creates safety gaps with poor ToC [21]. In addition, implementing PFE may require different levels of effort depending on the type and level of PFE. However, if the effectiveness of different PFE interventions remains unclear, it is a difficult task for stakeholders to implement such complex PEF interventions in and across complex care settings (i.e., ToC along the oncological care pathway) [5, 45, 46]. To move the field forward, to establish evidence and to inform practitioners and healthcare leaders, the growing literature from different oncological populations and multiple PFE interventions needs to be systematically synthesized.

Previous reviews of PFE in oncology care have merely focused on specific oncological populations (e.g. prostate or gynecological cancer [47, 48]) or specific PFE interventions (e.g. nurse-led engagement, patient decision aids, or e-health [49–53]). Because of these constraints, comprehensive meta-analyses are missing. Furthermore, few of these reviews have focused on ToC after cancer treatment [48, 49, 51]. Although available reviews evaluated interventions for oncology transitions, these did not explicitly or exclusively address PFE [54–58]. In addition, an overview of the specific characteristics of PFE interventions in this context is lacking. An appraisal of study quality as well as intervention characteristics are invaluable to determine the effectiveness according to intervention characteristics, inform the design of effective PFE strategies in this population, and identify research gaps. To this end, we have an eminent lack in our current knowledge base regarding the overall and specific effectiveness of different levels and types of PFE on patient safety in ToC after cancer treatment.

Here we describe the protocol for a study that aims to systematically review and quantitatively synthesize the best available evidence on effects of different PFE interventions on ‘transitional patient safety’ after cancer treatment to further treatment, to follow-up care or no care. In particular, we will address the following primary (1) and secondary (2–4) questions:

1. What are the effects of PFE on patient safety in transitions of care after cancer treatment of patients with oncological diseases?
2. What are the effects of different types of PFE on transitional patient safety in this context?
3. What are the effects of different levels of PFE on transitional patient safety in this context?
4. What are typical characteristics of PFE interventions that have already been studied for their effectiveness in this context?

## Methods

This protocol is reported in accordance with Preferred Reporting Items for Systematic Reviews and Meta-Analysis Protocols (PRISMA-P) [59] (cf., Table S1.1 in S1 File). The final report will follow PRISMA 2020 [60] and, if applicable, PRISMA harms [61] and PRISMA of complex interventions (PRISMA-CI) [62]. Where possible, we followed the methodological recommendation by the Cochrane Collaboration [63–65]. In order to increase transparency and visibility, a preliminary registration of the protocol has been made in PROSPERO (CRD42024546938) and OSF (doi.org/10.17605/OSF.IO/9XAMU).

### Eligibility criteria

Included studies will be required to be published in English and German (as speaking languages of authors) in peer-reviewed, scientific journals. Other inclusion criteria will follow the Participants, Interventions, Comparators, Outcomes and Study design (PICOS) framework [66]. All eligibility criteria are summarized in Table 1.

**Table 1:** Inclusion and exclusion criteria for study selection.

PICOS	Inclusion	Exclusion
Population and setting	• suffering or recovering from any form of cancer • in or after transition between cancer treatment and ◾ further treatment, ◾ follow-up care, or ◾ no care (e.g., discharge to home) • irrespective of the type of care (outpatient/inpatient), the medical or surgical specialty, type of cancer center or hospital	• no discernible transition after cancer treatment • transition of care prior to cancer treatment, e.g. from diagnosis to treatment
Intervention	• PFE at the level of direct care, team, organizational, institutional or managed-consortium, • all types of PFE along the continuum (consultation, involvement, partnership and shared leadership) • must be carried out for the purpose of a least one of the following aspects: ◾ improvement of interaction, cooperation, and communication, ◾ improvement of patient safety, ◾ improvement of PFE • no restrictions on age, sex, ethnicity or medical conditions and diseases of interventions participants • starts during cancer treatment, during or after post-treatment transition (within five years); activities that involve patient representatives or are not carried out directly with hospitalized patients can be carried out at any time	• PFE with the only objective of improving patient’s well-being • PFE at the policy-making level • unidirectional PFE activities directed only at patients • target population of the intervention consists solely of healthcare providers, without any PFE before or during the intervention • PFE activity starts later than five years after post-treatment transition, unless directed to patient representatives • PFE is implemented as a co-intervention and not as the focal component of the intervention
Comparators	(a) no control group (b) non-PFE (no intervention, standard practice control group, active intervention without PFE) (c) PFE (active intervention control group with another PFE strategy)	
Outcomes	• healthcare utilization, patient harms, adherence to treatment plan, patient experiences on transition safety • measured at baseline (i.e. before intervention) and at least once after the end of the intervention	• relevant outcomes being measured solely before the ToC of interest • outcomes of interest are measured qualitatively • only secondary outcomes of interests are measured and reported, not the primary outcomes
Study designs	• prospective, longitudinal intervention studies, including controlled and uncontrolled designs	• retrospective data collection or analyses • cross-sectional studies, case studies, case series
Others	• English or German language • peer-reviewed scientific journal	• conference abstracts, editorials, statements, and letters

Notes. PFE–patient and family engagement; further information can be found in the main text under ‘eligibility criteria’

#### Population and setting

Surveyed patient groups of interest will be patients regardless of age, sex, and ethnicity, suffering or recovering from any form of cancer. Patients must be undergoing current treatment, or after transition between cancer treatment (e.g., chemotherapy, immunotherapy, radiotherapy, oncological surgery) and any other cancer-related intervention (e.g., radiotherapy after surgery), follow-up care (defined as medical care once the cancer treatment has been completed [14]), or no care (i.e., discharge to home). Eligible investigations need to capture transition processes, regardless of type of care (i.e., outpatient or inpatient), medical or surgical specialty, type of cancer center or hospital where prior treatment and care were provided.

We will exclude investigations where no discernible transition after cancer treatment is surveyed. If it is unclear, whether transition after cancer treatment were scrutinized, we will contact corresponding authors, respectively. If there is no response, we will discuss the decision in the study team, including an oncology expert (author: ISW). Studies that include the populations of interest only as a subset of another population will only be considered if they report the relevant parameters for the participants of interest separately.

#### Intervention

In the literature, PFE has been described as an umbrella term for different concepts of active collaborations (see definition above) [28–30]. Moreover, reported definitions varied according to stakeholders’ multi-dimensional perspectives [28, 29, 67]. Hence, a variety of PFE activities across the continuum of engagement described by Carman et al. [30] and the healthcare system will be eligible. For this systematic review, PFE interventions must be carried out for the purpose of a least one of the following aspects:

- Improvement of interaction, cooperation, and communication between healthcare providers and patients or their relatives,
- Improvement of patient safety in terms of patient harm, adverse events, mortality, infection, healthcare utilization, medication adherence, personal patient safety experience, continuity of care,
- Improvement of patient and family engagement.

Consistent with our criteria, the nature and elements of PFE interventions may for example include self-management or self-care interventions, cognitive behavioral therapy, psychoeducation, lifestyle interventions, e-health interventions, speak up, structured surveys (e.g. implementing patient reported outcomes (PROMs) or other quality improvement efforts (e.g. PFE in the development of survivorship care plans). As PFE interventions are often applied across populations at an organizational level or may engage family members, there will be no restrictions on age, sex, ethnicity, medical conditions or diseases. Participants of the interventions can be, for example, patients themselves, family members, patient advocates or patient organizations, e.g. self-help groups or patient and family advisory boards.

The PFE activity can start during cancer treatment, during or after post-treatment transition (yet, maximally within five years). PFE activities that involve patient representatives or are not carried out directly with hospitalized patients can be carried out at any time. Trials with interventions that included another intervention in addition to PFE (co-intervention) will also be included, as long as PFE is the focal component of the intervention (e.g. trials that explicitly provide patient navigation or patient-centered care and use PFE as a subcomponent of these interventions are excluded). This assessment will be based on the objectives of the interventions in each trial and on discussion within the study team.

Concerning our non-eligibility criteria, we will exclude studies that solely perform PFE

- in course of individual treatments with the only objective of improving patient’s health status and/or well-being, such as quality of life, distress, satisfaction, or physical function;
- in the sense of more narrowly defined concepts such as patient activation [28] or non-meaningful patient engagement [68] by excluding unidirectional activities directed only at patients, without giving them the opportunity to express their views and be listened to [31, 68], e.g. free available educational material or knowledge transfer interventions;
- at the policy making level (e.g., changes of national legislation), as individual healthcare facilities or professionals themselves are not able to directly influence external circumstances and legal requirements in the care process;
- with the target population of the intervention being healthcare providers (e.g. training of healthcare providers on how to engage patients), without any engagement of patients or families before or during the intervention.

#### Comparators

Studies with none or any comparator will be included and broadly classified as follows [65]: (a) no control group, (b) non-PFE: overall; no intervention, standard practice or usual care control group (as defined by study authors), active intervention without PFE, or (c) PFE (active intervention control group with another PFE strategy).

#### Outcomes

Outcomes of interest are related to patient safety in transitions after cancer treatment. In the absence of a defined core outcome set for oncological ToC, we propose the following outcome categories: (1) primary outcome domains of interest are healthcare utilization, patient harm, adherence to treatment plan, patient experiences on transition safety, and (2) secondary outcome domains of interest are quality of life and distress. It is important to note that we will investigate the impact of PFE on patient harms, not the potential harms that may occur as a consequence or during the course of the intervention. Altogether, in literature reported problems in oncological ToC and some areas of cancer-related core outcome set [69] will be captured. The specific outcomes for extraction are listed in the ‘outcomes and prioritization’ section (Table 2).

**Table 2:** Hierarchical list of outcomes of interest in order of preference*.

	Outcome domains	Outcome measures
**Primary outcomes**	Healthcare utilization	1. Disease specific readmissions
		2. All-cause readmissions
		3. Emergency department visits
		4. Visits of outpatient healthcare service
	Patient harm^a^	1. Adverse events (if extractable: unspecified as well as specified, e.g. medical errors, medication safety events, infections, severe pain episodes: as well as avoidable vs. non-avoidable adverse events, if reported)
		2. Mortality
	Adherence to treatment or survivorship care plan	1. Measurement of the drug concentration in body fluids
		2. Electronic monitoring devices (e.g. electronic pill box, activity tracker)
		3. Standardized self-reports (Questionnaires) a. Medication Adherence Report Scale (MARS) b. Brief Adherence Rating Scale (BARS) c. Others
		4. Others
	Patient experiences and satisfaction on ‘transitional safety’ Patient reported—*We define this as the patient experience between the end of one cancer treatment and the start of another treatment*, *follow-up care or no care in terms of quality and safety from the patient’s perspective*.	1. Partner at Care Transition Measure (PACT-M)
		2. Care Transition Measure (CTM)
		3. Readiness for Hospital Discharge Scale (RHDS)
		4. Medical Home Care Coordination Survey (MHCCS)
		5. Continuity of Care Questionnaire (CCCQ)
		6. Others
	Other patient safety outcomes	Not previously defined^b^
**Secondary outcomes**	Wellbeing and quality of life Patient reported—*“refers to the health aspects of quality of life*, *generally considered to reflect the impact of disease and treatment on disability and daily functioning”* [79]	1. European Organization for Research and Treatment of Cancer Quality of Life Questionnaire Version 3.0 (EORTC QLQ-C30)
		2. The 36-Item Short Form Health Survey questionnaire (SF-36)
		3. Others
	Distress	1. Distress Thermometer
		2. Others

Notes.

* If several measures of an outcome domain are used and the respective effect measures are reported, results will be extracted for the highest preferred instrument; This preliminary list may be supplemented by other study-specific tools or patient safety outcomes as appropriate; Outcomes are grouped for synthesis at the level of outcome domains if the data are sufficiently homogeneous. Otherwise, a separate synthesis will be undertaken for each outcome measure; ^a^ no prior definition/list of the methods of outcome measure, as these are expected to be very heterogeneous; ^b^ As we expect a heterogeneous set of outcomes, we will also consider other patient safety outcomes reported in the included trials that we have not previously defined.

Eligible studies need to measure outcomes quantitatively at baseline (i.e., before intervention) and at least once after completion of intervention (i.e., at least one follow-up). We will exclude studies that measured the relevant outcomes solely prior to ToC of interest. Studies will also be excluded if they did not measure any of the outcomes of interest, but not if they measured them and only did not report them (see sensitivity analyses, missing outcome data). We exclude trials that only measure our secondary outcomes of interest, but not the primary outcomes.

### Study design and publication types

Controlled and uncontrolled study designs with longitudinal, prospective data collection will be eligible. This includes: Randomized controlled trials (RCTs; individual or variants like cluster or crossover designs), quasi-RCTs, controlled and uncontrolled before-after or interrupted time series studies, regardless of sample size. As we seek to obtain first, yet preliminary evidence on the impact of organizational PFE (as fewer RCTs are expected at this level based on prior scoping of the literature), and to examine harms [70], uncontrolled, longitudinal studies will also be eligible. Cross-sectional studies, case (series) studies, or studies with a retrospective design or analyses will be excluded. Also, conference abstracts, editorials, statements, and letters will be excluded.

### Information sources

We will follow PRISMA extension for Reporting Literature Searches in Systematic Reviews (PRISMA-S) [71] (Table S1.2 in S1 File). A comprehensive electronic database search of MEDLINE (via PubMed), EMBASE (via Ovid), CENTRAL (via Cochrane Library), CINAHL (via EBSCOhost), and APA PsycInfo (via EBSCOhost) from database inception to the date of search in 2024 will be conducted (versions of indexing databases will be documented if paid add-ons are used). Additionally, forward and backward citation searching of prior relevant reviews of the last five years (Table S2.6 in S2 File) and final included studies will be performed via the R Shiny app citationchaser [72, 73]. The WHO International Clinical Trials Registry Platform (ICTRP) and ClinicalTrials.gov will be searched to identify ongoing and completed, but unpublished trials. In order to identify further relevant projects and authors (exploratively; using keywords in an unsystematic way), dissertation databases (ProQuest, The Networked Digital Library of Theses and Dissertations), Google Scholar and the websites of the following pre-identified organizations will be searched: Institute for Patient and Family-Centered Care (IPFCC), Patient-Centered Outcomes Research Institute (PCORI), Agency for Healthcare Research and Quality (AHRQ) and Canadian Patient Safety Institute. Leading researchers in the field will be contacted by email to identify relevant trials missed in the database and grey literature searches.

### Search strategy

We will combine search terms for ‘oncology’ with terms for ‘patient and family engagement’ and ‘transitional patient safety’ with the following limits: Embase (records from Embase and Preprints, remove MEDLINE records), CINAHL (exclude MEDLINE records). No other filters or limitations will be used. The search strategy was initially developed for MEDLINE and subsequently modified for the other databases (Tables S2.1 to S2.5 in S2 File). A librarian from the University of Bonn was consulted in preparation and finalization of the search strategy.

### Selection process

We will use Covidence systematic reviews software (Veritas Health Innovation, Melbourne, Australia; www.covidence.org) to find and de-duplicate references and for the screening process. Three assessors will independently screen titles, abstracts and full-texts in duplicate and check them against the eligibility criteria (Table 1). For both screening steps, we will pilot the exclusion criteria in advance using 100 title and abstracts and 10% of the full-texts. Inter-rater reliability will be calculated. Disagreements will be resolved with a third assessor (MW or ISW) if necessary. All included references will be imported to the reference management software Zotero (version 7.0.0; Corporation for Digital Scholarship, Vienna, VA) to check for retracted publications using Zotero’s Retraction Watch integration. We will create a PRISMA flow diagram to document the included and excluded references for each step and the reasons for the full-text exclusions.

### Data collection process

Three independent assessors will extract the data using Covidence and Microsoft Excel (Version 2312; Microsoft Corporation, Redmond, USA). Study characteristics will be extracted by two reviewers (50% each), relevant outcome data by three reviewers in duplicate. We will set up extraction instructions and extraction table in advance and pilot testing them based on 10% of the included studies. Extracted data will be assessed for differences automatically in Covidence, inter-extractor-reliability will be calculated for primary outcomes. Potential disagreements and inconsistencies will be discussed with a third reviewer (MW or ISW). If it is not possible to collect the required data, information will be requested from the corresponding author at least three times over a three-week period.

### Data items and extraction

We will extract relevant publication information (author, title, year, journal, country, etc.), study methods (e.g. study aim, study design, recruitment and sampling procedures, source of funding) and participants’ demographics (e.g. age, sex, cancer disease characteristics, number), including key information on study eligibility criteria. In addition, we will collect detailed characteristics of PFE interventions (e.g. level and type, components, timing and duration), description of co-interventions, and definition and components of comparisons. Interventions will be additionally characterized using the template for intervention description and replication (TIDieR) [74]. For synthesis and also to address the secondary research questions, we will group the interventions according to the framework of Carman et al. [30]: PFE interventions (overall, broad); (a) levels of PFE: (a.1) direct care, (a.2) organizational design; (b) types of PFE: (b.1) consultation, (b.2) involvement, (b.3) partnership and shared leadership. If too few trials are identified for synthesis, b.2 and b.3 will be combined, also because these are not always clearly definable. Intervention control groups will be grouped as mentioned above (see comparators). If sufficient data and trials are available, we will split the individual interventions from comparator groups (b) and (c).

For outcomes of interest, we will collect information on the measurement tool (i.e. name/source, definition, whether a high or low score is favorable), specific metrics (e.g. post-intervention score, changes in score, presence of outcome (yes/no)), methods of aggregation and timing of outcome measurement, and results for each (sub-)group and each outcome at each time point (i.e. number of participants enrolled, allocated and included in the analysis, summary data for each group, between-group or within-group estimates and their precision). We expect the reported study data being diverse, so we pre-determine an order of preference for data extraction, following the recommendations of Daly et al.—for continuous data (e.g. quality of life) [75]: 1. mean difference (MD), standard error (SE) from ANCOVA; 2. change-from-baseline (CFB) arms means, standard deviation (SD), N; 3. arm means, SD at baseline and follow-up, N, correlation etc.; for event data (e.g. healthcare utilization, harms) [76, 77]: 1. data from 2 x 2 table; 2. log odds ratio (OR)/log risk ratio (RR), SE or confidence interval (CI); 3. OR/RR and their 95% CI etc. The data extraction process for ordinal outcomes will depend on whether the scale is dichotomized or treated as a continuous outcome for analysis. This choice is influenced by the methodology used by the study authors and presentation of their data, respectively. Therefore, we will follow the recommendations [77] and will extract ordinal data in all reported forms and determine the form of data for analysis after reviewing all studies. If multiple adjusted estimates are reported (especially for n-RCT), we will choose that one that is judged to best minimize the risk of bias due to confounding [70].

If necessary, we will use standard equations to convert arm-based data (arm means, SD, N) to contrast-based data (MD, SE) or to convert other reported data (e.g. when values but not SE are reported) [75–77]. Where data are presented in a figure only, we will use ImageJ (https://imagej.nih.gov/ij/) to extract the values by measuring the length of the axes in pixels followed by the length of the relevant data of interest [78].

For duration until follow-up, we will identify end of cancer treatment, short-term (< 30 days), medium-term (30–90 days), long-term (3–12 months) and very long-term (12–60 months) assessments for outcomes. If there are multiple follow-ups within each time period, we will extract the follow-up closest to 30 days for short-term, 90 days for medium-term, 12 months for long-term, and 60 months for very long-term. If two time points are equally close to these follow-ups, we will extract the one that is furthest from baseline.

### Outcomes and prioritization

If several measures of an outcome domain are used and the respective effect measures are reported, results will be extracted for the highest preferred instrument. A hierarchical list of prominent outcomes for patient safety in care transitions to be extracted, is presented in Table 2. As we expect a heterogeneous set of outcomes, we will also consider other patient safety outcomes reported in the included trials that we have not previously defined. Outcomes are grouped for synthesis at the level of outcome domains if the data are sufficiently homogeneous. Otherwise, a separate synthesis will be undertaken for each outcome measure, respectively.

### Risk of bias in individual studies

To evaluate the methodology quality of included RCTs, two reviewers will use the Cochrane revised Collaboration Risk of Bias Tool (RoB2) [80]. With this tool we will examine potential bias from the randomization process, deviations from intended interventions, missing outcome data, measurement of the outcome and in the selection of the reported results for individually and cluster randomized trials for each outcome. The rating will be categorized as low, unclear or high RoB. The same reviewers will use the revised JBI critical appraisal tool, consisting of 9 questions, to assess potential bias in quasi-experimental studies [81]. We will not assess the RoB of uncontrolled trials. Instead, we will automatically consider uncontrolled trials to have a high RoB. Any disagreements will be discussed by the study team until a consensus is reached.

As part of our RoB evaluation, we will assess and code for selective reporting of statistically significant results and the level of detail provided for non-significant results. We will identify instances where only significant outcomes are reported, or where non-significant outcomes are summarized with generic statements such as ’p > 0.05’.

### Data synthesis

In preparation of this protocol, we used the Intervention Synthesis Questions (InSynQ) checklist [65] to develop our data synthesis methods. If data are available in a suitable form and trial characteristics sufficiently homogenous, we will conduct a meta-analysis of included studies. All statistical analyses will be performed with R statistical software (R version 4.3.2 (2023-10-31) RStudio, Inc.). We will stratify all analyses according to study design—RCT, non-RCT and single-arm studies. We will use the broad category of ‘PFE interventions’ (overall) as the basis for separate, pairwise comparisons with different control groups (see comparators) and, within these, stratify by the specific types of interventions where possible (see subgroup analyses). We set the following order of importance for pairwise comparisons in advance: 1) PFE (overall) vs. non-PFE (overall), 2) PFE (overall) vs. active interventions without PFE, 3) PFE (overall) vs. usual care/standard practice, 4) subgroup analyses of different PFE levels (a.1, a.2) and types (b.1, b.2, b.3) vs. non-PFE, and 5) subgroup analyses of different PFE types and levels vs. active interventions with another PFE strategy. As we assume most PFE interventions being multi-component, we will not perform separate analyses for co-interventions.

Due to expected heterogeneity in trial characteristics, we will perform a pairwise inverse-variance random effect meta-analysis to estimate the average effect of PFE [82]. For effect measures of continuous data, we will standardize different outcomes of the same construct to a common scale using an internal reference SD to compare and pool the results of each trial [75]. As recommended [83–86], we will use the Hartung-Knapp-Sidik-Jonkmann method [87, 88] to calculate the respective 95% CI of the pooled effect with the Paule-Mandel estimator to estimate the between-study variance (τ^2^) [89] (HKSJ method). If necessary, we will perform an ad hoc variance correction (HKSJ-VC) [85]. If there are more than 10 studies and if τ^2^ is greater than zero, we will calculate prediction intervals to analyze the distribution of the true effect sizes [86, 90–92]. In addition to τ^2^, we will assess heterogeneity between studies by visual inspection of forest plots and by the I^2^ statistics [93]. We will also consider a multi-level meta-analysis if the data are sufficient.

For binary data, we will transform effect measures (OR is preferred to RR [76]) of the individual trials to a logarithmic scale with their SE before performing meta-analysis [76, 83]. In the absence of zero events, we will use the same approach as for continuous effect measures [76, 83, 85]. In the case of zero events or sparse data, we will use the Mantel-Haenzel method [94] (fixed effect model) without zero cell correction, as recommended [76, 83], since the primary concern is to detect whether there is any signal of an effect in the sparse data, rather than to account for heterogeneity. If the data of trials with sparse data are reported appropriately, we will consider using individual patient data (IPS) from the 2x2 tables and generalized linear mixed models (GLMM) [95, 96]. However, as this is currently unclear, we will decide on the further analysis of the IPD post hoc.

If data for the same outcomes are presented as binary data in some studies and as continuous data in others, we will transform OR to the standardized mean difference (SMD) (or vice versa) to combine the data [83, 97]. We will also perform this transformation to SMD for the quality of evidence rating (see below and Tables S3.1 and S3.2 in S3 File).

We will use the approaches described by Higgins et al. to adjust the effect estimates from variants of randomized trials [98]. Where possible, we will calculate the average effect and the average event probability of single-arm trials.

To aid interpretation of the clinical importance, we will back-transform SMD (to OR or to the most commonly used scale) and will calculate an absolute risk difference for the pooled OR [99] to present the results in the summary of findings. As a conservative approach and especially when no comparison with existing observational studies is possible [100], we will consider common cut-off points of 0.20, 0.50 and 0.80 for SMD as representing a small, moderate and large effect [101]. However, if available, we will also use the results of observational studies and the reported minimal important differences of our patients involved in the protocol (“at least 1 patient less does not experience any harm or does not use a health service or at least one event less per patient”) as references for interpretation.

We will use a complete-case analysis (using only participants with available outcome data) for the primary meta-analysis [102]. We will set statistical significance for all quantitative analyses at p<0.05. We will use tables and forest plots to present the results of the statistical synthesis. If the studies and data are insufficient for meta-analysis (e.g. because characteristics of studies are too heterogeneous, or data are incompletely reported), we will follow the guideline on synthesis without meta-analysis (SWiM) [103]. Narrative synthesis will present the review findings descriptively in text, tables and figures. In addition, we will structure summaries, tables and figures according to intervention groups and within these according to the certainty of the evidence and methodological quality (RoB grouping).

### Subgroup and sensitivity analyses

If sufficient data are available, subgroup and sensitivity analyses will be performed for primary outcomes of interest. If at least 10 studies are included in a meta-analysis, the following subgroup meta-analyses will be performed via mixed-effects model [86, 104] to explore possible causes of heterogeneity and to address the secondary research questions: 1) levels of PFE interventions (a.1, a.2), 2) types of PFE interventions (b.1 to b.3), 3) characteristics of the patients (age, cancer type), 4) transition of care contexts, and 5) type of participants in the intervention (see above).

We will perform sensitivity analyses to test the robustness of pooled results regarding study characteristics and methodological quality by removing the small sample (the exact cut-off is determined after the data extraction has been completed) or high RoB studies. When pooled estimates are statistically significant, we will perform further sensitivity analysis with progressively stringent but plausible assumptions about missing outcome data [102]. For missing data in continuous outcomes, we will use the most appropriate strategy described by Guyatt et al. [102]; for missing data in binary outcomes, we will use informative missingness odds ratio (IMOR) [102, 105]. If required, we will also perform sensitivity analyses by exclusion of outliers. Studies would be considered outlying if their confidence interval does not overlap with the confidence interval of the pooled effect [106].

### Meta-bias(es)

The potential for publication bias will be analyzed using Funnel plots by graphical verification of asymmetry. Test for asymmetry will be performed when more than 10 studies are included in meta-analysis (e.g. Egger’s test for MD or Peters’ test for binary outcomes) [107–109].

### Confidence in cumulative evidence

The Grading of Recommendations, Assessment, Development and Evaluation (GRADE) approach [110] will be used to assess the certainty of the evidence of pairwise meta-analysis comparisons. We will rate the quality of a body of evidence as high, moderate, low or very low by considering the study design as the starting point for initial quality (randomized trials as high, observational studies as low) and using five criteria for downgrading the quality of evidence (RoB, inconsistency, indirectness, imprecision, publication bias) and three criteria for upgrading the quality of evidence (large effect, dose response, plausible residual confounding) [111]. For certainty rating, we will use the partially contextualized approach by using guiding principles of thresholds (SMD; 0.20, 0.50 and 0.80) as we are not aware of any empirical values for our context and outcomes of interest [112]. The thresholds for rating and the operationalization of the GRADE criteria are presented in the Tables S3.1 and S3.2 in S3 File.

### Patient and public involvement

In course of development, three professional patient representatives contributed to this study protocol. We used a closed recruitment strategy via a federal self-help association and one-time unstructured individual interviews (direct method) [113] without audio-visual recording and with anonymous documentation. The feedback provided by the representatives on research questions, population, interventions, and outcomes of interest was considered in the protocol. An ethical vote was not required, as the exchanges with patient representatives were informal and without recording or storage of data. Other data underlying the manuscript are freely available.

## Discussion

Given the serious health consequences of inadequate ToC after active cancer treatment in patients with oncological diseases and the promising potential of patient involvement in safeguarding care, it is vital to establish robust evidence on effectiveness of successful PFE interventions in this vulnerable group.

We deem one of the main contributions of our planned systematic review with meta-analysis is to provide a first and comprehensive overview of the effectiveness of different PFE interventions in oncology on different ‘transitional patient safety’ outcomes—along the spectrum of various PFE types and across different levels of care (direct care, organizational) [30, 31]. We plan to determine, whether different types of PFE are differently effective in this context and whether there are differences in effectiveness depending on the level at which PFE is implemented. In addition, the specific characteristics of each PFE intervention will be recorded in detail and taken into account when analyzing and interpreting the results [114]. We will also consider a range of critical care transitions across the cancer care continuum [4, 5], including different sectors, interdisciplinary care providers, and different types of care and monitoring (e.g., transitions between episodes of cancer disease, different facilities, or inpatient and outpatient settings). Therefore, we hypothesize that our review will substantially extent the current knowledge base through providing a comprehensive overview in the context of ToC after cancer treatment. Our findings will—in contrast to previous other reviews in this context [47–50, 52, 53]–allow reliable inferences beyond specific PFE interventions, specific contexts, or specific cancer populations.

This comprehensive review is intended to help stakeholders navigate the field of PFE [115]. This evidence may help stakeholders in the course of design and development of PFE interventions to weigh up their own resources and implement the most promising interventions depend on specific context and local needs. Our systematic synthesis of studies will also identify research gaps that can be addressed in future studies (e.g., robust designs to evaluate the effectiveness of organizational PFE).

### Strengths and limitations

This study protocol is designed to promote transparency of the research process and to provide detailed methodological interventions. In addition to the inclusion criteria discussed above, the strengths of this systematic review will be that it follows established reporting guidelines and methodological recommendations and uses comprehensive information sources as well as search strategies. At the same time, our search is limited by excluding publications other than those in English and German language. In addition, we expect a heterogeneous study base, so we may not have been able to anticipate all methods of data extraction, synthesis and analysis in advance. Although we consulted patient representatives in our study planning, we may not have included all potentially relevant patient-relevant outcomes.

### Conclusion

Despite the incredible advances in oncological treatments, significant challenges in seamless and safe delivery of care remain. Safety issues for patients and families occur especially in transitions of care across different specialists and services. We anticipate that this systematic review will further help to establish robust evidence on how patients and families can effectively engage in support of safer oncological care. Ultimately, our evidence synthesis will contribute to safeguarding oncological care services that meet the needs of patients and families.

### Statements

**Patient and public involvement:** Patients and/or the public were involved in the design of this protocol. Further details can be found in the methods section.

## Supporting information

S1 File PRISMA checklist. (DOCX)

S2 File Seach strategies. (DOCX)

S3 File Grading of Recommendations, Assessment, Development and Evaluation (GRADE). (DOCX)
