# Supplementary material for: The impact of patient engagement on patient safety in care transitions after cancer treatment: Protocol for a systematic review and meta-analysis
Source: PLoS One. 2024 Aug 27;19(8):e0307831. doi: 10.1371/journal.pone.0307831 (PMC11349088; doi:10.1371/journal.pone.0307831)
Supplement: S1 File — (DOCX) [file pone.0307831.s001.docx]

# S1. PRIMSA checklist.

### S1.1 Table. Preferred Reporting Items for Systematic Review and Meta-Analysis Protocols (PRISMA‑P) 2015 checklist [1]

| **Section and topic** | **Item No** | **Checklist item** | **Location where item is reported** |
| --- | --- | --- | --- |
| **ADMINISTRATIVE INFORMATION** | | | |
| **Title:** | | | |
| Identification | 1a | Identify the report as a protocol of a systematic review | p. 1; ll. 4-8 |
| Update | 1b | If the protocol is for an update of a previous systematic review, identify as such | NA |
| Registration | 2 | If registered, provide the name of the registry (such as PROSPERO) and registration number | p. 2; l. 50; p. 5; ll. 125, 126 |
| **Authors:** | | | |
| Contact | 3a | Provide name, institutional affiliation, e-mail address of all protocol authors; provide physical mailing address of corresponding author | p. 1 |
| Contributions | 3b | Describe contributions of protocol authors and identify the guarantor of the review | Submission system |
| Amendments | 4 | If the protocol represents an amendment of a previously completed or published protocol, identify as such and list changes; otherwise, state plan for documenting important protocol amendments | NA |
| **Support:** | | | |
| Sources | 5a | Indicate sources of financial or other support for the review | p. 23 |
| Sponsor | 5b | Provide name for the review funder and/or sponsor | p. 23; ll. 493-496 |
| Role of sponsor or funder | 5c | Describe roles of funder(s), sponsor(s), and/or institution(s), if any, in developing the protocol | p. 23; ll. 493-496 |
| **INTRODUCTION** | | | |
| Rationale | 6 | Describe the rationale for the review in the context of what is already known | pp. 3-5 |
| Objectives | 7 | Provide an explicit statement of the question(s) the review will address with reference to participants, interventions, comparators, and outcomes (PICO) | p. 5; ll. 113-118 |
| **METHODS** | | | |
| Eligibility criteria | 8 | Specify the study characteristics (such as PICO, study design, setting, time frame) and report characteristics (such as years considered, language, publication status) to be used as criteria for eligibility for the review | pp. 6-10 |
| Information sources | 9 | Describe all intended information sources (such as electronic databases, contact with study authors, trial registers or other grey literature sources) with planned dates of coverage | pp. 10, 11 |
| Search strategy | 10 | Present draft of search strategy to be used for at least one electronic database, including planned limits, such that it could be repeated | p. 11; S2 |
| **Study records:** | | | |
| Data management | 11a | Describe the mechanism(s) that will be used to manage records and data throughout the review | pp. 11; 12; ll. 248-262 |
| Selection process | 11b | State the process that will be used for selecting studies (such as two independent reviewers) through each phase of the review (that is, screening, eligibility, and inclusion in meta-analysis) | pp. 11; 12; ll. 248-259 |
| Data collection process | 11c | Describe planned method of extracting data from reports (such as piloting forms, done independently, in duplicate), any processes for obtaining and confirming data from investigators | p. 12; ll. 261-269 |
| Data items | 12 | List and define all variables for which data will be sought (such as PICO items, funding sources), any pre-planned data assumptions and simplifications | pp. 12-14; ll. 270-315 |
| Outcomes and prioritization | 13 | List and define all outcomes for which data will be sought, including prioritization of main and additional outcomes, with rationale | pp. 14, 15; ll. 316-331, Table 2 |
| Risk of bias in individual studies | 14 | Describe anticipated methods for assessing risk of bias of individual studies, including whether this will be done at the outcome or study level, or both; state how this information will be used in data synthesis | pp. 15, 16; ll. 332-345 |
| Data synthesis | 15a | Describe criteria under which study data will be quantitatively synthesised | pp. 16-18 |
|  | 15b | If data are appropriate for quantitative synthesis, describe planned summary measures, methods of handling data and methods of combining data from studies, including any planned exploration of consistency (such as I^2^, Kendall’s τ) | pp. 16-18; ll. 361-401 |
|  | 15c | Describe any proposed additional analyses (such as sensitivity or subgroup analyses, meta-regression) | pp. 18, 19; ll. 408-425 |
|  | 15d | If quantitative synthesis is not appropriate, describe the type of summary planned | p. 18; ll. 402-407 |
| Meta-bias(es) | 16 | Specify any planned assessment of meta-bias(es) (such as publication bias across studies, selective reporting within studies) | p. 19; ll. 426-429 |
| Confidence in cumulative evidence | 17 | Describe how the strength of the body of evidence will be assessed (such as GRADE) | p. 19, 20; ll. 430-441 |

Note. l.- line(s);

[1] Moher D, Shamseer L, Clarke M, Ghersi D, Liberati A, Petticrew M, et al. Preferred reporting items for systematic review and meta-analysis protocols (PRISMA-P) 2015 statement. Syst Rev. 2015;4: 1. doi:10.1186/2046-4053-4-1

**S1.2 Table. Preferred Reporting Items for Systematic Reviews and Meta-Analyses extension for Reporting Literature Searches in Systematic Reviews (PRISMA-S) Checklist [2]**

| **Topic** | **No** | **Item** | **Location where item is reported** |
| --- | --- | --- | --- |
| **INFORMATION SOURCES AND METHODS** | | | |
| Database name | 1 | Name each individual database searched, stating the platform for each. | p. 10; ll. 225-227 |
| Multi-database searching | 2 | If databases were searched simultaneously on a single platform, state the name of the platform, listing all of the databases searched. | p. 10; ll. 225-227 |
| Study registries | 3 | List any study registries searched. | p. 10; ll. 231-233 |
| Online resources and browsing | 4 | Describe any online or print source purposefully searched or browsed (e.g., tables of contents, print conference proceedings, web sites), and how this was done. | pp. 10, 11; ll. 233-238 |
| Citation searching | 5 | Indicate whether cited references or citing references were examined, and describe any methods used for locating cited/citing references (e.g., browsing reference lists, using a citation index, setting up email alerts for references citing included studies). | p. 10; ll. 229-231 |
| Contacts | 6 | Indicate whether additional studies or data were sought by contacting authors, experts, manufacturers, or others. | p. 11; ll. 238-240 |
| Other methods | 7 | Describe any additional information sources or search methods used. | pp. 10, 11; 233-236 |
| **SEARCH STRATEGIES** | | | |
| Full search strategies | 8 | Include the search strategies for each database and information source, copied and pasted exactly as run. | p. 11; ll. 241-247; S2 |
| Limits and restrictions | 9 | Specify that no limits were used, or describe any limits or restrictions applied to a search (e.g., date or time period, language, study design) and provide justification for their use. | p. 11; ll. 244-246; S2 |
| Search filters | 10 | Indicate whether published search filters were used (as originally designed or modified), and if so, cite the filter(s) used. | p. 11; ll. 244-246; S2 |
| Prior work | 11 | Indicate when search strategies from other literature reviews were adapted or reused for a substantive part or all of the search, citing the previous review(s). | NA |
| Updates | 12 | Report the methods used to update the search(es) (e.g., rerunning searches, email alerts). | NA |
| Dates of searches | 13 | For each search strategy, provide the date when the last search occurred. | NA (protocol) |
| **PEER REVIEW** | | | |
| Peer review | 14 | Describe any search peer review process. | p. 11; ll. 246, 247 |
| **MANAGING RECORDS** | | | |
| Total Records | 15 | Document the total number of records identified from each database and other information sources. | NA (protocol) |
| Deduplication | 16 | Describe the processes and any software used to deduplicate records from multiple database searches and other information sources. | p. 11; ll. 249-251 |

Note. l.- line(s);

[2] Rethlefsen ML, Kirtley S, Waffenschmidt S, Ayala AP, Moher D, Page MJ, et al. PRISMA-S: an extension to the PRISMA Statement for Reporting Literature Searches in Systematic Reviews. Systematic Reviews. 2021;10: 39. doi:10.1186/s13643-020-01542-z
