# Supplementary material for: The impact of patient engagement on patient safety in care transitions after cancer treatment: Protocol for a systematic review and meta-analysis
Source: PLoS One. 2024 Aug 27;19(8):e0307831. doi: 10.1371/journal.pone.0307831 (PMC11349088; doi:10.1371/journal.pone.0307831)
Supplement: S2 File — (DOCX) [file pone.0307831.s002.docx]

# S2 Tables. Search strategies.

### S2.1 Table. MEDLINE (via Pubmed)

| **Search** | **Query** |
| --- | --- |
| #1 | "oncology"[Title/Abstract] OR "oncogen*"[Title/Abstract] OR "cancer"[Title/Abstract] OR "chemotherapy"[Title/Abstract] OR "neoplasm*"[Title/Abstract] OR "carcinogen*"[Title/Abstract] OR "carcinom*"[Title/Abstract] OR "tumor"[Title/Abstract] OR "sarcoma"[Title/Abstract] OR "metastas*"[Title/Abstract] OR "melanom*"[Title/Abstract] OR "lymphom*"[Title/Abstract] OR "leukemi*"[Title/Abstract] OR "abnormal cell proliferation"[Title/Abstract] OR "maligna*"[Title/Abstract] OR "Carcinoma"[MeSH Terms] OR "Neoplasms"[MeSH Terms] OR "Medical Oncology"[MeSH Terms] OR "Surgical Oncology"[MeSH Terms] OR "Integrative Oncology"[MeSH Terms] OR "Radiation Oncology"[MeSH Terms] OR "Psycho-Oncology"[MeSH Terms] OR "Oncology Service, Hospital"[MeSH Terms] OR "Cancer Care Facilities"[MeSH Terms] OR "Cancer Survivors"[MeSH Terms] OR "Cancer pain"[MeSH Terms] |
| #2 | "engagement"[Title/Abstract] OR "involvement"[Title/Abstract] OR "participation"[Title/Abstract] OR "empowerment"[Title/Abstract] OR "family interaction"[Title/Abstract] OR "patient interaction"[Title/Abstract] OR "patient support"[Title/Abstract] OR "self management"[Title/Abstract] OR "patient initiated"[Title/Abstract] OR "self care"[Title/Abstract] OR "patient advocacy"[Title/Abstract] OR "patient advis*"[Title/Abstract] OR "patient and family advisory councils"[Title/Abstract] OR "PFAC"[Title/Abstract] OR "patient survey"[Title/Abstract: ~2] OR "self-help"[Title/Abstract] OR "supportive care"[Title/Abstract] OR "community-based coordination"[Title/Abstract: ~2] OR "postdischarge follow-up"[Title/Abstract: ~1] OR “family caregiver”[Title/Abstract] OR “lay caregiver” [Title/Abstract] OR "Social Participation"[MeSH Terms] OR "Community Participation"[MeSH Terms] OR "Empowerment"[MeSH Terms] OR "Self Care"[MeSH Terms] OR "Self-management"[MeSH Terms] OR "Patient Advocacy"[MeSH Terms] OR “Caregivers”[MeSH Terms] |
| #3 | "healthcare utilisation"[Title/Abstract] OR "healthcare utilization"[Title/Abstract] OR "medical service usage"[Title/Abstract] OR "medical resource usage"[Title/Abstract] OR "re-admission"[Title/Abstract] OR "readmission"[Title/Abstract] OR "rehospitalization"[Title/Abstract] OR "return hospital"[Title/Abstract] OR "hospital revisits"[Title/Abstract] OR "reattendance"[Title/Abstract] OR "re-attendance"[Title/Abstract] OR "care coordination"[Title/Abstract] OR "coordination of care"[Title/Abstract] OR "after-care"[Title/Abstract] OR "aftercare"[Title/Abstract] OR "follow-up care"[Title/Abstract] OR "survivorship care"[Title/Abstract: ~2] OR "Patient Readmission"[MeSH Terms] OR "Continuity of Patient Care"[MeSH Terms] |
| #4 | “patient safety"[Title/Abstract] OR "safety monitoring"[Title/Abstract] OR "mortality"[Title/Abstract] OR "death"[Title/Abstract] OR "harm"[Title/Abstract] OR "adverse events"[Title/Abstract] OR "adverse incidents"[Title/Abstract] OR "adverse reactions"[Title/Abstract] OR "undesirable effect*"[Title/Abstract] OR "medical error"[Title/Abstract] OR "medication error*"[Title/Abstract] OR "quality of life"[Title/Abstract] OR "satisfaction"[Title/Abstract] OR "experience"[Title/Abstract] OR “distress”[Title/Abstract] OR "adherence"[Title/Abstract] OR "Quality of Life"[MeSH Terms] OR "Medical errors"[MeSH Terms] OR "Mortality"[MeSH Terms] OR "Patient satisfaction"[MeSH Terms] OR "Patient harm"[MeSH Terms] |
| #5 | "coordination of care"[Title/Abstract] OR "care coordination"[Title/Abstract] OR "discharge*"[Title/Abstract] OR "postdischarge"[Title/Abstract] OR "transition"[Title/Abstract] OR "handoff"[Title/Abstract] OR "change over"[Title/Abstract] OR "patient discharge"[MeSH Terms] OR "Continuity of Patient Care"[MeSH Terms] OR "healthcare utilisation"[Title/Abstract] OR "healthcare utilization"[Title/Abstract] OR "medical service usage"[Title/Abstract] OR "medical resource usage"[Title/Abstract] OR "re-admission"[Title/Abstract] OR "readmission"[Title/Abstract] OR "rehospitalization"[Title/Abstract] OR "hospitalization"[Title/Abstract] OR "return hospital"[Title/Abstract] OR "hospital revisits"[Title/Abstract] OR "reattendance"[Title/Abstract] OR "re-attendance"[Title/Abstract] OR "Patient Readmission"[MeSH Terms] OR "after-care"[Title/Abstract] OR "aftercare"[Title/Abstract] OR "follow-up care"[Title/Abstract] OR “survivorship care"[Title/Abstract: ~2] |
| #6 | #4 AND #5 |
| #7 | #3 OR #6 |
| #8 | #1 AND #2 AND #7 |

### S2.2 Table. EMBASE (via Ovid)

| **Search** | **Query** |
| --- | --- |
| #1 | (oncology or oncogen* or cancer or chemotherapy or neoplasm* or carcinogen* or carcinom* or tumor or sarcoma or metastas* or melanom* or lymphom* or leukemi* or "abnormal cell proliferation" or maligna*).ab,ti. or exp Carcinoma/ or exp Neoplasms/ or exp "Medical Oncology"/ or exp "Surgical Oncology"/ or exp "Integrative Oncology"/ or exp "Radiation Oncology"/ or exp Psycho-Oncology/ or exp "Oncology Service, Hospital"/ or exp "Cancer Care Facilities"/ or exp "Cancer Survivors"/ or exp "Cancer pain"/ |
| #2 | (engagement or involvement or participation or empowerment or "family interaction" or "patient interaction" or "patient support" or "self management" or "patient initiated" or "self care" or "patient advocacy" or "patient advis*" or "patient and family advisory councils" or PFAC or (patient adj3 survey) or self-help or "supportive care" or (community-based adj3 coordination) or (postdischarge adj2 follow-up) or “family caregiver” or “lay caregiver”).ab,ti. or exp "Social Participation"/ or exp "Community Participation"/ or exp Empowerment/ or exp "Self Care"/ or exp Self-management/ or exp "Patient Advocacy"/ or exp”Caregivers”/ |
| #3 | ("healthcare utilisation" or "healthcare utilization" or "medical service usage" or "medical resource usage" or re-admission or readmission or rehospitalization or "return hospital" or "hospital revisits" or reattendance or re-attendance or "care coordination" or "coordination of care" or after-care or aftercare or "follow-up care" or (survivorship adj3 care)).ti,ab. or exp "Patient Readmission"/ or exp "Continuity of Patient Care"/ |
| #4 | ("patient safety" or "safety monitoring" or mortality or death or harm or "adverse events" or "adverse incidents" or "adverse reactions" or "undesirable effect*" or "medical error" or "medication error*" or "quality of life" or satisfaction or experience or distress or adherence).ti,ab. or exp "Quality of Life"/ or exp "Medical errors"/ or exp Mortality/ or exp "Patient satisfaction"/ or exp "Patient harm"/ |
| #5 | ("coordination of care" or "care coordination" or discharge* or postdischarge or transition or handoff or "change over" or after-care or aftercare or "follow-up care" or (survivorship adj3 care) or "healthcare utilisation" or "healthcare utilization" or "medical service usage" or "medical resource usage" or re-admission or readmission or rehospitalization or hospitalization or "return hospital" or "hospital revisits" or reattendance or re-attendance).ti,ab. or exp "patient discharge"/ or exp "Continuity of Patient Care"/ or exp "Patient Readmission"/ |
| #6 | #4 AND #5 |
| #7 | #3 OR #6 |
| #8 | #1 AND #2 AND #7, Limits: Records from Embase and Preprints, Remove MEDLINE Records |

### S2.3 Table. CENTRAL (via Cochrane Library)

| **Search** | **Query** |
| --- | --- |
| #1 | oncology:ti,ab OR oncogen*:ti,ab OR cancer:ti,ab OR chemotherapy:ti,ab OR neoplasm*:ti,ab OR carcinogen*:ti,ab OR carcinom*:ti,ab OR tumor:ti,ab OR sarcoma:ti,ab OR metastas*:ti,ab OR melanom*:ti,ab OR lymphom*:ti,ab OR leukemi*:ti,ab OR "abnormal cell proliferation":ti,ab OR maligna*:ti,ab OR [mh Carcinoma] OR [mh Neoplasms] OR [mh "Medical Oncology"] OR [mh "Surgical Oncology"] OR [mh "Integrative Oncology"] OR [mh "Radiation Oncology"] OR [mh “Psycho-Oncology”] OR [mh "Oncology Service, Hospital"] OR [mh "Cancer Care Facilities"] OR [mh "Cancer Survivors"] OR [mh "Cancer pain"] |
| #2 | engagement:ti,ab OR involvement:ti,ab OR participation:ti,ab OR empowerment:ti,ab OR "family interaction":ti,ab OR "patient interaction":ti,ab OR "patient support":ti,ab OR "self management":ti,ab OR "patient initiated":ti,ab OR "self care":ti,ab OR "patient advocacy":ti,ab OR (patient NEXT advis*):ti,ab OR "patient and family advisory councils":ti,ab OR PFAC:ti,ab OR (patient NEXT/2 survey):ti,ab OR "self help":ti,ab OR "supportive care":ti,ab OR ("community based" NEXT/2 coordination):ti,ab OR (postdischarge NEXT/1 "follow up"):ti,ab OR “lay caregiver”:ti,ab OR “family caregiver”:ti,ab OR [mh "Social Participation"] OR [mh "Community Participation"] OR [mh Empowerment] OR [mh "Self Care"] OR [mh “Self-management”] OR [mh "Patient Advocacy"] OR [mh “Caregivers”] |
| #3 | "healthcare utilisation":ti,ab OR "healthcare utilization":ti,ab OR "medical service usage":ti,ab OR "medical resource usage":ti,ab OR "re admission":ti,ab OR readmission:ti,ab OR rehospitalization:ti,ab OR "return hospital":ti,ab OR "hospital revisits":ti,ab OR reattendance:ti,ab OR "re attendance":ti,ab OR "care coordination":ti,ab OR "coordination of care":ti,ab OR "after care":ti,ab OR aftercare:ti,ab OR "follow up care":ti,ab OR (survivorship NEXT/2 care):ti,ab OR [mh "Patient Readmission"] OR [mh "Continuity of Patient Care"] |
| #4 | "patient safety":ti,ab OR "safety monitoring":ti,ab OR mortality:ti,ab OR death:ti,ab OR harm:ti,ab OR "adverse events":ti,ab OR "adverse incidents":ti,ab OR "adverse reactions":ti,ab OR (undesirable NEXT effect*):ti,ab OR "medical error":ti,ab OR (medication NEXT error*):ti,ab OR "quality of life":ti,ab OR satisfaction:ti,ab OR experience:ti,ab OR distress:ti,ab OR adherence:ti,ab OR [mh "Quality of Life"] OR [mh "Medical errors"] OR [mh Mortality] OR [mh "Patient satisfaction"] OR [mh "Patient harm"] |
| #5 | "coordination of care":ti,ab OR "care coordination":ti,ab OR discharge*:ti,ab OR postdischarge:ti,ab OR transition:ti,ab OR handoff:ti,ab OR "change over":ti,ab OR [mh "patient discharge"] OR [mh "Continuity of Patient Care"] OR "healthcare utilisation":ti,ab OR "healthcare utilization":ti,ab OR "medical service usage":ti,ab OR "medical resource usage":ti,ab OR "re admission":ti,ab OR readmission:ti,ab OR rehospitalization:ti,ab OR hospitalization:ti,ab OR "return hospital":ti,ab OR "hospital revisits":ti,ab OR reattendance:ti,ab OR "re attendance":ti,ab OR [mh "Patient Readmission"] OR "after care":ti,ab OR aftercare:ti,ab OR "follow up care":ti,ab OR (survivorship NEXT/2 care):ti,ab |
| #6 | #4 AND #5 |
| #7 | #3 OR #6 |
| #8 | #1 AND #2 AND #7 |

### S2.4 Table. CINAHL (via EBSCOhost)

| **Search** | **Query** |
| --- | --- |
| #1 | (TI oncology OR AB oncology) OR (TI oncogen* OR AB oncogen*) OR (TI cancer OR AB cancer) OR (TI chemotherapy OR AB chemotherapy) OR (TI neoplasm* OR AB neoplasm*) OR (TI carcinogen* OR AB carcinogen*) OR (TI carcinom* OR AB carcinom*) OR (TI tumor OR AB tumor) OR (TI sarcoma OR AB sarcoma) OR (TI metastas* OR AB metastas*) OR (TI melanom* OR AB melanom*) OR (TI lymphom* OR AB lymphom*) OR (TI leukemi* OR AB leukemi*) OR (TI "abnormal cell proliferation" OR AB "abnormal cell proliferation") OR (TI maligna* OR AB maligna*) OR (MH Carcinoma) OR (MH Neoplasms) OR (MH "Medical Oncology") OR (MH "Surgical Oncology") OR (MH "Integrative Oncology") OR (MH "Radiation Oncology") OR (MH Psycho-Oncology) OR (MH "Oncology Service, Hospital") OR (MH "Cancer Care Facilities") OR (MH "Cancer Survivors") OR (MH "Cancer pain") |
| #2 | (TI engagement OR AB engagement) OR (TI involvement OR AB involvement) OR (TI participation OR AB participation) OR (TI empowerment OR AB empowerment) OR (TI "family interaction" OR AB "family interaction") OR (TI "patient interaction" OR AB "patient interaction") OR (TI "patient support" OR AB "patient support") OR (TI "self management" OR AB "self management") OR (TI "patient initiated" OR AB "patient initiated") OR (TI "self care" OR AB "self care") OR (TI "patient advocacy" OR AB "patient advocacy") OR (TI "patient advis*" OR AB "patient advis*") OR (TI "patient and family advisory councils" OR AB "patient and family advisory councils") OR (TI PFAC OR AB PFAC) OR (TI patient w2 survey OR AB patient w2 survey) OR (TI self-help OR AB self-help) OR (TI "supportive care" OR AB "supportive care") OR (TI community-based w2 coordination OR AB community-based w2 coordination) OR (TI postdischarge w1 follow-up OR AB postdischarge w1 follow-up) OR (TI “family caregiver” OR AB “family caregiver”) OR (TI “lay caregiver” OR AB “lay caregiver”) OR (MH "Social Participation") OR (MH "Community Participation") OR (MH Empowerment) OR (MH "Self Care") OR (MH Self-management) OR (MH "Patient Advocacy") OR (MH “Caregivers”) |
| #3 | (TI "healthcare utilisation" OR AB "healthcare utilisation") OR (TI "healthcare utilization" OR AB "healthcare utilization") OR (TI "medical service usage" OR AB "medical service usage") OR (TI "medical resource usage" OR AB "medical resource usage") OR (TI re-admission OR AB re-admission) OR (TI readmission OR AB readmission) OR (TI rehospitalization OR AB rehospitalization) OR (TI "return hospital" OR AB "return hospital") OR (TI "hospital revisits" OR AB "hospital revisits") OR (TI reattendance OR AB reattendance) OR (TI re-attendance OR AB re-attendance) OR (TI "care coordination" OR AB "care coordination") OR (TI "coordination of care" OR AB "coordination of care") OR (TI after-care OR AB after-care) OR (TI aftercare OR AB aftercare) OR (TI "follow-up care" OR AB "follow-up care") OR (TI survivorship w2 care OR AB survivorship w2 care) OR (MH "Patient Readmission") OR (MH "Continuity of Patient Care") |
| #4 | (TI "patient safety" OR AB "patient safety") OR (TI "safety monitoring" OR AB "safety monitoring") OR (TI mortality OR AB mortality) OR (TI death OR AB death) OR (TI harm OR AB harm) OR (TI "adverse events" OR AB "adverse events") OR (TI "adverse incidents" OR AB "adverse incidents") OR (TI "adverse reactions" OR AB "adverse reactions") OR (TI "undesirable effect*" OR AB "undesirable effect*") OR (TI "medical error" OR AB "medical error") OR (TI "medication error*" OR AB "medication error*") OR (TI "quality of life" OR AB "quality of life") OR (TI satisfaction OR AB satisfaction) OR (TI experience OR AB experience) OR (TI distress OR AB distress) OR (TI adherence OR AB adherence) OR (MH "Quality of Life") OR (MH "Medical errors") OR (MH Mortality) OR (MH "Patient satisfaction") OR (MH "Patient harm") |
| #5 | (TI "coordination of care" OR AB "coordination of care") OR (TI "care coordination" OR AB "care coordination") OR (TI discharge* OR AB discharge*) OR (TI postdischarge OR AB postdischarge) OR (TI transition OR AB transition) OR (TI handoff OR AB handoff) OR (TI "change over" OR AB "change over") OR (MH "patient discharge") OR (MH "Continuity of Patient Care") OR (TI "healthcare utilisation" OR AB "healthcare utilisation") OR (TI "healthcare utilization" OR AB "healthcare utilization") OR (TI "medical service usage" OR AB "medical service usage") OR (TI "medical resource usage" OR AB "medical resource usage") OR (TI re-admission OR AB re-admission) OR (TI readmission OR AB readmission) OR (TI rehospitalization OR AB rehospitalization) OR (TI hospitalization OR AB hospitalization) OR (TI "return hospital" OR AB "return hospital") OR (TI "hospital revisits" OR AB "hospital revisits") OR (TI reattendance OR AB reattendance) OR (TI re-attendance OR AB re-attendance) OR (MH "Patient Readmission") OR (TI after-care OR AB after-care) OR (TI aftercare OR AB aftercare) OR (TI "follow-up care" OR AB "follow-up care") OR (TI survivorship w2 care OR AB survivorship w2 care) |
| #6 | #4 AND #5 |
| #7 | #3 OR #6 |
| #8 | #1 AND #2 AND #7, Limits: Exclude MEDLINE records |

### S2.5 Table. APA PsycInfo (via EBSCOhost)

| **Search** | **Query** |
| --- | --- |
| #1 | (TI oncology OR AB oncology) OR (TI oncogen* OR AB oncogen*) OR (TI cancer OR AB cancer) OR (TI chemotherapy OR AB chemotherapy) OR (TI neoplasm* OR AB neoplasm*) OR (TI carcinogen* OR AB carcinogen*) OR (TI carcinom* OR AB carcinom*) OR (TI tumor OR AB tumor) OR (TI sarcoma OR AB sarcoma) OR (TI metastas* OR AB metastas*) OR (TI melanom* OR AB melanom*) OR (TI lymphom* OR AB lymphom*) OR (TI leukemi* OR AB leukemi*) OR (TI "abnormal cell proliferation" OR AB "abnormal cell proliferation") OR (TI maligna* OR AB maligna*) OR (MH Carcinoma) OR (MH Neoplasms) OR (MH "Medical Oncology") OR (MH "Surgical Oncology") OR (MH "Integrative Oncology") OR (MH "Radiation Oncology") OR (MH Psycho-Oncology) OR (MH "Oncology Service, Hospital") OR (MH "Cancer Care Facilities") OR (MH "Cancer Survivors") OR (MH "Cancer pain") |
| #2 | (TI engagement OR AB engagement) OR (TI involvement OR AB involvement) OR (TI participation OR AB participation) OR (TI empowerment OR AB empowerment) OR (TI "family interaction" OR AB "family interaction") OR (TI "patient interaction" OR AB "patient interaction") OR (TI "patient support" OR AB "patient support") OR (TI "self management" OR AB "self management") OR (TI "patient initiated" OR AB "patient initiated") OR (TI "self care" OR AB "self care") OR (TI "patient advocacy" OR AB "patient advocacy") OR (TI "patient advis*" OR AB "patient advis*") OR (TI "patient and family advisory councils" OR AB "patient and family advisory councils") OR (TI PFAC OR AB PFAC) OR (TI patient w2 survey OR AB patient w2 survey) OR (TI self-help OR AB self-help) OR (TI "supportive care" OR AB "supportive care") OR (TI community-based w2 coordination OR AB community-based w2 coordination) OR (TI postdischarge w1 follow-up OR AB postdischarge w1 follow-up) OR (TI “family caregiver” OR AB “family caregiver”) OR (TI “lay caregiver” OR AB “lay caregiver”) OR (MH "Social Participation") OR (MH "Community Participation") OR (MH Empowerment) OR (MH "Self Care") OR (MH Self-management) OR (MH "Patient Advocacy") OR (MH “Caregivers”) |
| #3 | (TI "healthcare utilisation" OR AB "healthcare utilisation") OR (TI "healthcare utilization" OR AB "healthcare utilization") OR (TI "medical service usage" OR AB "medical service usage") OR (TI "medical resource usage" OR AB "medical resource usage") OR (TI re-admission OR AB re-admission) OR (TI readmission OR AB readmission) OR (TI rehospitalization OR AB rehospitalization) OR (TI "return hospital" OR AB "return hospital") OR (TI "hospital revisits" OR AB "hospital revisits") OR (TI reattendance OR AB reattendance) OR (TI re-attendance OR AB re-attendance) OR (TI "care coordination" OR AB "care coordination") OR (TI "coordination of care" OR AB "coordination of care") OR (TI after-care OR AB after-care) OR (TI aftercare OR AB aftercare) OR (TI "follow-up care" OR AB "follow-up care") OR (TI survivorship w2 care OR AB survivorship w2 care) OR (MH "Patient Readmission") OR (MH "Continuity of Patient Care") |
| #4 | (TI "patient safety" OR AB "patient safety") OR (TI "safety monitoring" OR AB "safety monitoring") OR (TI mortality OR AB mortality) OR (TI death OR AB death) OR (TI harm OR AB harm) OR (TI "adverse events" OR AB "adverse events") OR (TI "adverse incidents" OR AB "adverse incidents") OR (TI "adverse reactions" OR AB "adverse reactions") OR (TI "undesirable effect*" OR AB "undesirable effect*") OR (TI "medical error" OR AB "medical error") OR (TI "medication error*" OR AB "medication error*") OR (TI "quality of life" OR AB "quality of life") OR (TI satisfaction OR AB satisfaction) OR (TI experience OR AB experience) OR (TI distress OR AB distress) OR (TI adherence OR AB adherence) OR (MH "Quality of Life") OR (MH "Medical errors") OR (MH Mortality) OR (MH "Patient satisfaction") OR (MH "Patient harm") |
| #5 | (TI "coordination of care" OR AB "coordination of care") OR (TI "care coordination" OR AB "care coordination") OR (TI discharge* OR AB discharge*) OR (TI postdischarge OR AB postdischarge) OR (TI transition OR AB transition) OR (TI handoff OR AB handoff) OR (TI "change over" OR AB "change over") OR (MH "patient discharge") OR (MH "Continuity of Patient Care") OR (TI "healthcare utilisation" OR AB "healthcare utilisation") OR (TI "healthcare utilization" OR AB "healthcare utilization") OR (TI "medical service usage" OR AB "medical service usage") OR (TI "medical resource usage" OR AB "medical resource usage") OR (TI re-admission OR AB re-admission) OR (TI readmission OR AB readmission) OR (TI rehospitalization OR AB rehospitalization) OR (TI hospitalization OR AB hospitalization) OR (TI "return hospital" OR AB "return hospital") OR (TI "hospital revisits" OR AB "hospital revisits") OR (TI reattendance OR AB reattendance) OR (TI re-attendance OR AB re-attendance) OR (MH "Patient Readmission") OR (TI after-care OR AB after-care) OR (TI aftercare OR AB aftercare) OR (TI "follow-up care" OR AB "follow-up care") OR (TI survivorship w2 care OR AB survivorship w2 care) |
| #6 | #4 AND #5 |
| #7 | #3 OR #6 |
| #8 | #1 AND #2 AND #7 |

### S2.6 Table. Citation Searches of Relevant Reviews

| **Search** | **Citation searches** |
| --- | --- |
| #1 | Forward and backward citation tracking of prior relevant reviews of the last five years [3–17] |

**References of the S2.6 Table**

3. Bonetti L, Tolotti A, Anderson G, Nania T, Vignaduzzo C, Sari D, et al. Nursing interventions to promote patient engagement in cancer care: A systematic review. Int J Nurs Stud. 2022;133: 104289. doi:10.1016/j.ijnurstu.2022.104289

4. Tomlinson J, Cheong V-L, Fylan B, Silcock J, Smith H, Karban K, et al. Successful care transitions for older people: a systematic review and meta-analysis of the effects of interventions that support medication continuity. Age Ageing. 2020;49: 558–569. doi:10.1093/ageing/afaa002

5. Kershaw VF, Chainrai M, Radley SC. Patient initiated follow up in Obstetrics and Gynaecology: A systematic review. Eur J Obstet Gynecol Reprod Biol. 2022;272: 123–129. doi:10.1016/j.ejogrb.2022.02.181

6. Martínez-González NA, Plate A, Markun S, Senn O, Rosemann T, Neuner-Jehle S. Shared decision making for men facing prostate cancer treatment: a systematic review of randomized controlled trials. Patient Prefer Adherence. 2019;13: 1153–1174. doi:10.2147/PPA.S202034

7. Husebø ALM, Søreide JA, Kørner H, Storm M, Wathne HB, Richardson A, et al. eHealth interventions to support colorectal cancer patients’ self-management after discharge from surgery—an integrative literature review. Support Care Cancer. 2023;32: 11. doi:10.1007/s00520-023-08191-7

8. Ziegler E, Hill J, Lieske B, Klein J, dem Knesebeck O von, Kofahl C. Empowerment in cancer patients: Does peer support make a difference? A systematic review. Psycho-Oncology. 2022;31: 683–704. doi:10.1002/pon.5869

9. Di Nitto M, Sollazzo F, Biagioli V, Pucciarelli G, Torino F, Alvaro R, et al. Self-care behaviors in patients with cancer treated with oral anticancer agents: a systematic review. Support Care Cancer. 2022;30: 8465–8483. doi:10.1007/s00520-022-07166-4

10. Chan RJ, Milch VE, Crawford-Williams F, Agbejule OA, Joseph R, Johal J, et al. Patient navigation across the cancer care continuum: An overview of systematic reviews and emerging literature. CA: A Cancer Journal for Clinicians. 2023;73: 565–589. doi:10.3322/caac.21788

11. Liang H, Tao L, Ford EW, Beydoun MA, Eid SM. The patient-centered oncology care on health care utilization and cost: A systematic review and meta-analysis. Health Care Management Review. 2020;45: 364. doi:10.1097/HMR.0000000000000226

12. Høeg BL, Bidstrup PE, Karlsen RV, Friberg AS, Albieri V, Dalton SO, et al. Follow-up strategies following completion of primary cancer treatment in adult cancer survivors. Cochrane Database Syst Rev. 2019;2019: CD012425. doi:10.1002/14651858.CD012425.pub2

13. Bucknall TK, Hutchinson AM, Botti M, McTier L, Rawson H, Hitch D, et al. Engaging patients and families in communication across transitions of care: An integrative review. Patient Education and Counseling. 2020;103: 1104–1117. doi:10.1016/j.pec.2020.01.017

14. Becker C, Zumbrunn S, Beck K, Vincent A, Loretz N, Müller J, et al. Interventions to Improve Communication at Hospital Discharge and Rates of Readmission: A Systematic Review and Meta-analysis. JAMA Netw Open. 2021;4: e2119346. doi:10.1001/jamanetworkopen.2021.19346

15. Tyler N, Hodkinson A, Planner C, Angelakis I, Keyworth C, Hall A, et al. Transitional Care Interventions From Hospital to Community to Reduce Health Care Use and Improve Patient Outcomes: A Systematic Review and Network Meta-Analysis. JAMA Network Open. 2023;6: e2344825. doi:10.1001/jamanetworkopen.2023.44825

16. Oksholm T, Gissum KR, Hunskår I, Augestad MT, Kyte K, Stensletten K, et al. The effect of transitions intervention to ensure patient safety and satisfaction when transferred from hospital to home health care-A systematic review. J Adv Nurs. 2023;79: 2098–2118. doi:10.1111/jan.15579

17. O’Donnell R, Savaglio M, Skouteris H, Banaszak-Holl J, Moranl C, Morris H, et al. The Effectiveness of Transition Interventions to Support Older Patients From Hospital to Home: A Systematic Scoping Review. J Appl Gerontol. 2021;40: 1628–1636. doi:10.1177/0733464820968712
