# Supplementary material for: The impact of patient engagement on patient safety in care transitions after cancer treatment: Protocol for a systematic review and meta-analysis
Source: PLoS One. 2024 Aug 27;19(8):e0307831. doi: 10.1371/journal.pone.0307831 (PMC11349088; doi:10.1371/journal.pone.0307831)
Supplement: S3 File — (DOCX) [file pone.0307831.s003.docx]

# S3 Tables. Grading of Recommendations, Assessment, Development and Evaluation (GRADE). [18]

### Table S3.1 Effect thresholds for rating imprecision and inconsistency [19]

| **Threshold*** | **Magnitude of effect** |
| --- | --- |
| \|SMD\| ≤ 0.2 | Trivial |
| 0.2 < \|SMD\| < 0.5 | Small |
| 0.5 < \|SMD\| ≤ 0.8 | Moderate |
| \|SMD\| > 0.8 | Large |

Notes. |SMD| - absolute values of the standardized mean difference; * if SMD are available or are possible to calculate otherwise we will use the null effect-threshold [20,21].

### Table S3.2 Operationalization of Grading of Recommendations, Assessment, Development and Evaluation (GRADE) criteria [18,22], partially adopted from the Confidence in Network Meta-Analysis (CINeMA) approach [23]

| **GRADE criteria** | **Operationalization** |
| --- | --- |
| Study limitations (risk of bias) | Downgrade one level:   - high risk of bias across most of the body of available evidence [24] |
| Publication bias  [25] | Downgrade one level if at least one of the following scenarios apply:   - body of evidence is mainly based on a number of small studies with positive early findings (e.g. pilot studies) - small studies with industry funding across most of the body of available evidence - evidence of funnel plot asymmetry and statistical tests |
| Imprecision  [20,21,26] | Downgrade one level:   - 95% CI crosses one SMD-threshold^a^ // 95% CI crosses null effect minimal^b^   Downgrade two levels if one of the following scenarios apply:   - 95% CI crosses two SMD-thresholds^a^ // 95% CI crosses null effect substantially^b^ - If the OIS is considered (this is the case when we use the null effect-threshold and the 95% CI does not cross the null effect and the effect is sufficiently large):   - ratio of the upper and lower boundary of the 95% CI is more than 2.5 (for odds ratio) or 3.0 (for risk ratio)   - total sample size of the meta-analysis is smaller than 30-50% of the OIS   Downgrade three levels:   - 95% crosses three SMD-thresholds^a^ |
| Inconsistency  [23,27,28] | If prediction intervals are calculated:  Downgrade one level if one of the following scenarios apply:   - no imprecision and 95% CI crosses null effect into opposite area of trivial^a^ - 95% CI crosses null effect minimal^b^ - one level imprecision and 95% PI crosses null effect end extends beyond opposite area of trivial^a^ // one level imprecision and 95% PI crosses null effect substantially^b^   Downgrade two levels:   - 95% CI crosses no threshold and 95% PI crosses two thresholds^a^ // 95% PI crosses null effect substantially^b^   If prediction intervals are not calculated:  Downgrade one level if one of the following scenarios apply:   - point estimates lie substantially on either side of the null-effect threshold - minimal or no overlap between 95% CI intervals of primary studies - visual inspection of forest plots and τ^2^ indicate heterogeneity   Downgrade two levels:   - none of the pre-planned subgroup analyses explain heterogeneity |
| Indirectness  [29] | Downgrade one level:   - if the differences are considered sufficient to make a difference in outcome likely   Downgrade two levels:   - the body of evidence is mainly based on studies without direct comparisons (i.e. single arm trials)* |

Notes. CI – confidence interval, OIS - optimal information size, PI – prediction interval, ^a^ - when using SMD-thresholds, ^b^- when using the null effect-threshold, * we see usual care as a direct comparison

**References of the S3 Tables**

18. Guyatt G, Oxman AD, Akl EA, Kunz R, Vist G, Brozek J, et al. GRADE guidelines: 1. Introduction—GRADE evidence profiles and summary of findings tables. Journal of Clinical Epidemiology. 2011;64: 383–394. doi:10.1016/j.jclinepi.2010.04.026

19. Cohen J. Statistical power analysis for the behavioral sciences. Routledge; 2013.

20. Zeng L, Brignardello-Petersen R, Hultcrantz M, Mustafa RA, Murad MH, Iorio A, et al. GRADE Guidance 34: update on rating imprecision using a minimally contextualized approach. J Clin Epidemiol. 2022;150: 216–224. doi:10.1016/j.jclinepi.2022.07.014

21. Schünemann HJ, Neumann I, Hultcrantz M, Brignardello-Petersen R, Zeng L, Murad MH, et al. GRADE guidance 35: update on rating imprecision for assessing contextualized certainty of evidence and making decisions. J Clin Epidemiol. 2022;150: 225–242. doi:10.1016/j.jclinepi.2022.07.015

22. Balshem H, Helfand M, Schünemann HJ, Oxman AD, Kunz R, Brozek J, et al. GRADE guidelines: 3. Rating the quality of evidence. Journal of Clinical Epidemiology. 2011;64: 401–406. doi:10.1016/j.jclinepi.2010.07.015

23. Nikolakopoulou A, Higgins JPT, Papakonstantinou T, Chaimani A, Del Giovane C, Egger M, et al. CINeMA: An approach for assessing confidence in the results of a network meta-analysis. PLoS Med. 2020;17: e1003082. doi:10.1371/journal.pmed.1003082

24. Guyatt GH, Oxman AD, Vist G, Kunz R, Brozek J, Alonso-Coello P, et al. GRADE guidelines: 4. Rating the quality of evidence—study limitations (risk of bias). Journal of Clinical Epidemiology. 2011;64: 407–415. doi:10.1016/j.jclinepi.2010.07.017

25. Guyatt GH, Oxman AD, Montori V, Vist G, Kunz R, Brozek J, et al. GRADE guidelines: 5. Rating the quality of evidence—publication bias. Journal of Clinical Epidemiology. 2011;64: 1277–1282. doi:10.1016/j.jclinepi.2011.01.011

26. Guyatt GH, Oxman AD, Kunz R, Brozek J, Alonso-Coello P, Rind D, et al. GRADE guidelines: 6. Rating the quality of evidence—imprecision. Journal of Clinical Epidemiology. 2011;64: 1283–1293. doi:10.1016/j.jclinepi.2011.01.012

27. Guyatt GH, Oxman AD, Kunz R, Woodcock J, Brozek J, Helfand M, et al. GRADE guidelines: 7. Rating the quality of evidence—inconsistency. Journal of Clinical Epidemiology. 2011;64: 1294–1302. doi:10.1016/j.jclinepi.2011.03.017

28. Guyatt G, Zhao Y, Mayer M, Briel M, Mustafa R, Izcovich A, et al. GRADE guidance 36: updates to GRADE’s approach to addressing inconsistency. J Clin Epidemiol. 2023;158: 70–83. doi:10.1016/j.jclinepi.2023.03.003

29. Guyatt GH, Oxman AD, Kunz R, Woodcock J, Brozek J, Helfand M, et al. GRADE guidelines: 8. Rating the quality of evidence--indirectness. J Clin Epidemiol. 2011;64: 1303–1310. doi:10.1016/j.jclinepi.2011.04.014
